# A 12-month study of dialectical behavioral therapy for bοrderline patients suffering from eating disorders

**DOI:** 10.1007/s40519-023-01612-w

**Published:** 2023-10-05

**Authors:** Efi Liakopoulou, Georgia Vassalou, Chara Tzavara, Fragiskos Gonidakis

**Affiliations:** 1 1st Psychiatric Department, Eginition Hospital, National and Kapodistrian University of Athens, Athens, Greece; 2 Medical School, National and Kapodistrian University of Athens, Athens, Greece

**Keywords:** Borderline personality disorder, Eating disorders, Dialectical behavioral therapy, Anorexia nervosa, Bulimia nervosa

## Abstract

**Purpose:**

Individuals with eating disorders (ED) and comorbid borderline personality disorder (BPD) may benefit from therapies focusing on emotion regulation, such as dialectical behavioral therapy (DBT). The aim of the study was to evaluate the effectiveness of one-year standard DΒΤ enhanced with cognitive-behavioral therapy (CBT) strategies for patients suffering from ED and BPD.

**Methods:**

Seventy-two BPD and ED (anorexia and bulimia nervosa) participants were recruited from the eating disorders unit of the 1st Psychiatric Department of National and Kapodistrian University of Athens. All participants completed one year of standard DBT. ED-related behaviors were added to the treatment plan according to the DBT targeting hierarchy. Individual therapy and skills training group sessions were adapted to incorporate CBT strategies for nutritional and weight restoration. BPD and ED symptomatology were measured at the beginning and at the end of one year of treatment.

**Results:**

The major finding of the study was the significant improvement of patients in all the outcome measurements after one year of treatment. The study's second finding was that the severity of BPD symptomatology was significantly related to the severity of ED symptomatology. It was also shown that improvement of the patients coping skills was correlated with the reduction of ED and BPD symptomatology.

**Conclusions:**

These results support previous studies on the effectiveness of DBT for comorbid BPD and EDs. Despite the promising results, randomized controlled trials are needed to establish the efficacy of DBT for BPD and ED patients.

**Level of evidence:**

Level IV: Evidence obtained from multiple time series with or without the intervention, such as case studies. Dramatic results in uncontrolled trials might also be regarded as this type of evidence.

**Supplementary Information:**

The online version contains supplementary material available at 10.1007/s40519-023-01612-w.

## Introduction

Eating disorders (ED) are a group of psychiatric disorders characterized by disordered eating or weight control behaviors that lead to altered food consumption or absorption [1]. Comorbidity is quite common among patients with anorexia nervosa (AN), bulimia nervosa (BN) and binge eating disorder (BED), with 80% of sufferers meeting diagnostic criteria for other disorders besides EDs at some point in their life [2]. Α meta-analysis examining the prevalence of personality disorders among individuals with EDs demonstrated that the most common comorbid personality disorder in patients with EDs is borderline personality disorder (BPD), with prevalence rates of 28.4% in individuals with BN, 25.5% in individuals with AN-binge-eating/purging subtype (AN-BP), 10.8% in individuals with AN-restrictive subtype (AN-R), and 11.7% in those with BED [3]. The relationship between BPD symptoms and EDs is complex, as BPD symptoms can act as a predisposing or severity factor of EDs [4, 5]. While the mechanisms underlying this comorbidity and the implications for treatment are still under investigation, patients with EDs and coexisting BPD symptoms are considered a “difficult to treat” clinical group [6].

The comorbidity of EDs and BPD has been associated with higher levels of anxiety, depression, more severe general psychopathology and life-threatening behaviors, lower expectations regarding sufferers’ ability to regulate affect and finally, greater difficulty in reducing ED symptoms than individuals with EDs alone [7–9]. It has been reported that BPD with co-occurring BN is associated with a higher risk of repeated suicide attempts, BPD with co-occurring AN is associated with an increased risk of repeated non-suicidal self-harm, and BPD with co-occurring BED is associated with a higher rate of other Axis I disorders in addition to EDs [8].

Considering the course of the comorbid disorders, there are studies reporting no adverse effects of BPD on the severity of ED symptoms or treatment outcomes [10–12], while a follow-up study of the course of EDs in individuals who were also diagnosed with BPD found that although the initial ED regressed at the 10-year follow-up, there were fluctuations in symptomatology with migration to other EDs [13].

It is critical to understand the differences between patients with EDs and BPD and patients without such comorbidity in order to ensure that appropriately targeted interventions are provided to this high-risk group [6]. Affective instability, a key characteristic of BPD which is also observed in EDs, should be prioritized when treating patients with ED and BPD [14]. Individuals with high affective instability, such as BPD patients, may use eating related maladaptive behaviors, such as binging and purging in order to regulate negative emotions and rapid mood fluctuations [14, 15]. Consequently, these patients may benefit from therapies focusing on emotion regulation and distress tolerance strategies, such as dialectical behavioral therapy (DBT) [7, 16].

Regarding EDs, cognitive-behavioral therapy (CBT) is considered a first-line treatment for BN and BED [17] while the “enhanced” form of the treatment (CBT-E) was found to be an effective “transdiagnostic” treatment for BN, EDNOS and possibly AN [18, 19]. CBT-E puts an emphasis on the overevaluation of body and weight for the individuals self-esteem and targets the perpetuating mechanisms of ED such as the effects of starvation or the rewarding nature of binge eating. The treatment targets are prioritized starting with weight and nutritional restoration and moving on to the dysfunctional beliefs and sustain chronic feelings of low esteem [18, 19].

Regarding BPD, DBT was initially developed to treat chronically suicidal individuals diagnosed with BPD using principles derived from behaviorism, Zen meditation and dialectical philosophy [20–22]. DPT has been proven to be effective as a treatment for BPD in several studies [23]. DBT has been adopted and applied in individuals suffering from ED disorders with quite promising results [24]. The effectiveness of DBT for the treatment of BPD and ED have been shown in a number of studies.

The first study evaluated a pilot program of DBT in women with BPD and BN or EDNOS and all individuals appeared to benefit from DBT regarding general functioning, dysfunctional eating behaviors, and self-harm at the 18-month follow-up. [25]. Some years later, a second study evaluated a 6-month DBT program in women who met the criteria for BPD and BN or BED and it was found significant improvements in the measures related to EDs and suicidal and non-suicidal self-injury behaviors up to 6 months after the completion of the treatment [26]. Furthermore, a 3-month adaptation of the DBT combined with an additional cognitive-behavioral module specific for EDs was evaluated in an open trial in a sample of 24 inpatients with BPD and EDs. The results showed that self-reported ratings of diet-related complaints, general psychopathology, and overall psychosocial functioning significantly improved after the treatment and at the 15-month follow-up [27]. Recently, in a naturalistic setting, a non-randomized controlled trial of DBT was compared to treatment as usual cognitive behavior therapy (TAU-CBT) for the comorbidity of BPD and EDs. Patients who received DBT showed significant reductions in dysfunctional behaviors associated with BPD which are used to regulate emotions, non-suicidal self-harm, and depressive symptoms, as well as a significant increase in cognitive performance and overall functioning [28]. Finally, the long-term outcomes (4- and 6-year post-treatment) of the above study were published some years later showing that individuals who received DBT had improved significantly in measurements of depression, emotional eating, anger, impulsivity, adaptability, and emotional expression at the 4- and 6-year post-treatment assessment compared to the pre-treatment assessment. However, comparing the two interventions did not result in significant differences over the follow-up years, suggesting that a new program such as DBT may be as effective as a well-established program such as TAU-CBT [29]. A recent meta-analysis, reported that DBT was found to produce the largest effects in reducing disordered eating behaviors among other third-wave interventions [30].

The aim of this study is to evaluate the effectiveness of 1-year standard DΒΤ enhanced with CBT strategies for weight and nutritional restoration for patients suffering from ED and BPD.

## Method

### Participants

Participants were recruited during the years 2017 to 2022 from the Eating Disorders Unit of the 1st Psychiatric Department of National and Kapodistrian University of Athens in Eginition Hospital. During their first appointment, all participants who met the diagnosis of both BPD and ED during the above-mentioned period of time were approached.

The diagnosis of BPD and ED was confirmed by a senior psychiatrist. Also, SCID-II was conducted during the second appointment by a psychologist [31]. Considering the ED diagnostic categories, due to the small sample size anorectic type ED that did not meet the full criteria for AN (BMI over 17, lack of weight phobia or body image distortion) were categorized as AN and the same applied to BN. Also the fact that the ED unit provides only group DBT skills training for BED prohibited the inclusion of patients suffering from BED in the study. The inclusion criteria were: BMI over 12 and/or absence of serious medical complication due to malnutrition, age between 17 and 35 years, adequate knowledge of the Greek language (read and write), lack of psychotic disorder, developmental disability and substance use disorder that required specialized treatment. It should be noted that considering substance use BPD patients that did not met the full criteria for substance use disorder were included in the study. In fact a large portion of the participants especially those suffering from BN reported that they were using some kind of psychoactive substance mainly cannabis, alcohol, nicotine and less often cocaine. All patients that were found to be suffering from BPD and ED were offered the same treatment option (DBT), and they were required to commit to one year of treatment.

### Measurements

All measurements with the exception of the demographics, were administered twice during the initial assessment and after the completion of one year of treatment. The questionnaires were administered by the primary researcher, who was not in any way involved in the patient’s treatment.

The following questionnaires were used:

- Borderline Symptom List 23 (BSL 23) [32]. The questionnaire has 23 items measuring BPD symptomatology during the last week prior to the investigation. The questionnaire has been translated, back-translated and adjusted in Greek. The Cronbach alpha was calculated at 0.95. BSL 23 includes also a supplementary list of 11 items for the assessment of behaviors often observed in individuals suffering from BPD, Item 4 and 5 refer to ED symptomatology (binge eating and purging behaviors).
- Ways of Coping Checklist (WCCL) [33]. The questionnaire assesses possible ways of managing crises during the last month prior to the investigation. Specifically, it investigates whether patients use DBT skills or dysfunctional coping mechanisms when faced with a psychological crisis or blame others for their predicament. The questionnaire has been translated, back-translated and adjusted in Greek. The Chronbach alpha was calculated at 0.94 for the DBT skills subscale, 0.76 for the general dysfunctional coping subscale, and 0.70 for the blaming subscale.
- Suicidal behaviors questionnaire (SBQ) [34]. The questionnaire is used for the measurement of suicidal ideation. It is a self-complementary questionnaire assessing suicide ideation, expectations for suicide, communications, threats regarding suicide, and suicidal behaviors. The questionnaire has been translated, back-translated and adjusted in Greek. The Chronbach alpha was calculated at 0.84.
- Eating Disorders Examination (EDE-Q) [35]. The questionnaire is used to assess ED symptomatology and related behaviors. It has four subscales measuring restraint eating, eating concern, weight concern, and body shape concern. The Greek version of the questionnaire has been validated by Pliatskidou et al. [36]. The Cronbach alpha was calculated at 0.92 for restraint eating, 0.88 for eating concern, 0.91 for shape concern, 0.88 for weight concern, and 0.92 for the total EDE-Q score.

### The treatment

All participants completed one year of standard DBT. Standard DBT involves weekly individual psychotherapy, skills training group, the therapists’ group consultation meeting, and access to 24-h telephone coaching. Eight therapists in total, four in each consultation group, provided the treatment (individual and skills training) to the participants. All therapists engaged in the treatment of the patients that were included in the study had been trained in DBT and CBT-E and had more than 5 years of clinical experience in DBT treatment for BPD and ED patients. The therapists were organized into two consultation groups that met on a weekly basis. All therapists were supervised every 2 weeks by one senior psychiatrist with more than 10 years of clinical and training experience in DBT for BPD and CBT for ED.

According to DBT binge eating was regarded as a short-term emotion regulation behavior, while caloric intake restraint was regarded as a long-term behavior resulting in the attenuation of intense emotions. ED-related behaviors were added to the treatment plan according to the DBT targeting hierarchy. Thus, top priority was given to behaviors that could threaten the individual's life (such as starvation, intense purging), followed by therapy-interfering behaviors (such as coming to therapy late, not filling in the food diary card). In contrast, behaviors that negatively impacted the individual's quality of life (such as binge eating) were coming third in priority. Individual therapy and skills training group sessions were adapted to incorporate CBT strategies for nutritional and weight restoration. Specifically, the patients were urged to keep a food diary that was reviewed during the weekly sessions. Weight measurement was also conducted at the first half of the session. CBT-E strategies for gradual nutritional and weight restoration such as establishing a five meals per day routine, increasing caloric intake, reducing specific food avoidance, and reduction of purging behaviors and compulsive exercising were used as described into the treatment manual [37]. Also, mindful eating and body awareness practice were used as part of the mindfulness skills training (non-judgmental body awareness, mindful eating). Finally, DBT distress tolerance skills for substance abstinence were taught and practiced with the clients targeting the food addiction behaviors observed in ED patients.

All participants were asked to have a complete medical check-up before engaging in the whole treatment process. During therapy, the individual therapist collaborated with the medical team that monitored the patient's medical status.

### Statistical analysis

Continuous variables were expressed as mean values (standard deviation, SD), while categorical variables were expressed as absolute and relative frequencies. Paired Student’s t-tests and Wilcoxon signed tests were used for time comparisons. Spearman correlation coefficients (rho) were used to explore the association of two continuous variables. All reported *p* values are two-tailed. Statistical significance was set at *p* < 0.05, and analyses were conducted using the Statistical Package for the Social Science (SPSS) software (version 26.0).

## Results

In total, 92 patients were assessed and found eligible to be included in the study. One patient refused to participate in the study. Of the 91 patients who started treatment and agreed to participate in the study, 15 dropped out before completing the first year of treatment. No statistical significance difference were found in the baseline measurements between the group that concluded the first year of treatment and the group that dropped out of treatment. Seventy-two (72) participants completed one year of treatment. 90,3% of them were women. Their mean age was 26.7 years old (SD = 6.7 years), and their mean BMI was 23.5 kg/m 2 (SD = 5.4 kg/m). Considering the ED diagnosis, 12 (16.7%) participants were suffering from AN binge/purge type or anorectic type ED and 60 (83.3%) were suffering from BN or bulimic type ED. The majority of the participants were unmarried (83.3%), and high school graduates (43.1%) while most of them had seen a mental health professional twice during their lifetime (40.4%) (Table 1).

**Table 1:** Demographic variables of the participants

	Patients (*N* = 72)
	*N* (%) or mean (SD)
Gender	
Men	7 (9.7)
Women	65 (90.3)
Age	26.7 (6.7)
Working status	
Employed full-time	14 (19.4)
Employed part-time	10 (13.9)
Unemployed	17 (23.6)
Student	27 (37.5)
Other	4 (5.6)
Family status	
Unmarried	60 (83.3)
Married	1 (1.4)
Married with children	3 (4.2)
Divorced–widowed	3 (4.2)
Living with partner	4 (5.6)
Unmarried with children	1 (1.4)
Educational level	
Middle school graduate	7 (9.7)
High school graduate	31 (43.1)
Technical University alumni	9 (12.5)
University alumni	7 (9.7)
Postgraduate degree	7 (9.7)
Other	11 (15.3)
Visits to a mental health professional during lifetime	
1	18 (31.6)
2	23 (40.4)
3	16 (28.1)

The means, standard deviations, and *p* values of the outcome measures at pre-treatment (1st measurement) and post-treatment (2nd measurement) are summarized in Table 2. Scores in BSL-23 and EDE-Q scales and the SBQ score diminished significantly at the second measurement. Furthermore, the DBT Skills subscale increased significantly at the second measurement, while the WCCL General dysfunctional coping factor decreased significantly. No significant change was observed with the WCCL blaming others factor.

**Table 2:** Comparison between the beginning and the end of one year of therapy

	1st measurement	2nd measurement	*P*
	Mean (SD)	Mean (SD)	
BSL-23 score	2.3 (1)	1.1 (0.6)	< 0.001++
EDE-Q			
Restraint	2.7 (2.2)	1.6 (1.5)	< 0.001++
Eating concern	2.5 (1.9)	1.3 (1.2)	< 0.001++
Shape concern	3.9 (1.8)	2.3 (1.4)	< 0.001++
Weight concern	3.5 (1.9)	1.9 (1.3)	< 0.001++
Global EDE-Q score	3.2 (1.8)	1.8 (1.3)	< 0.001++
DBT-WCCL			
DBT skills subscale	1.2 (0.5)	1.9 (0.3)	< 0.001+
General dysfunctional coping factor	2.2 (0.4)	1.5 (0.4)	< 0.001+
Blaming others factor	1.9 (0.6)	1.9 (0.4)	0.972+
SBQ score	16.5 (15.1)	7.7 (9.7)	< 0.001++

+paired-sample *t*-test; ++Wilcoxon test

Considering the diagnosis of ED, all scales were similar in both groups (AN and BN) at both measurements (before and after one year of treatment (*p* > 0.05). Due to the above, all patients were grouped for the statistical analysis of the results (Supplementary Table 1).

The BSL-23 supplementary behavioral assessment of the patients during the first and second measurements is summarized in Table 3. The quality of their overall status increased significantly at the second measurement, one year after DBT. Moreover, the frequency of reports from patients who hurt themselves, told others they wanted to commit suicide, binged, deliberately threw up, consciously acted dangerously, used substances, used unprescribed medication, and had rage outbreaks to others reduced significantly at the second measurement.

**Table 3:** BSL-23 supplementary behavioral assessment of the patients during the 1st and 2nd measurement. IQR: interquartile range

During last week:	1st measurement	2nd measurement	*P* +
	Median (IQR)	Median (IQR)	
Quality of your overall image	30 (20–50)	50 (40–60)	< 0.001
I hurt myself (cut myself, burned myself, drowned, hit my head on the wall, etc.)	0 (0–1)	0 (0–0)	< 0.001
I told other people that I wanted to commit suicide	0 (0–1)	0 (0–0)	< 0.001
I attempted suicide	0 (0–0)	0 (0–0)	0.109
I had episodes of binge eating	2 (0–3)	1 (0–2)	< 0.001
I induced vomiting	0 (0–2)	0 (0–0.5)	0.008
I consciously engaged in high-risk behaviors such as driving too fast, running on tall buildings’ roofs, balancing bridges, etc.	0 (0–1)	0 (0–0)	< 0.001
I got drunk	0 (0–1)	0 (0–1)	0.476
I used psychoactive substances	0 (0–1)	0 (0–0)	0.007
I have used a medicine not prescribed to me or, if prescribed, I took a dose higher than specified	0 (0–1)	0 (0–0)	0.001
I have had outbursts of uncontrollable anger or physically attacked others	1 (0–2)	0 (0–0)	< 0.001
I have had indiscriminate sexual encounters that I was ashamed of or made me feel angry afterward	0 (0–0)	0 (0–0)	0.058

+ Wilcoxon test

BSL-23 score was positively correlated with all four subscales of the EDE-Q score and the total EDE-Q score, indicating that more severe BPD symptomatology correlates to more severe ED symptomatology both at the beginning and at the end of one year of treatment (Table 4).

**Table 4:** Spearman’s correlation coefficients between EDE-Q and BSL-23 scales

	BSL-23 score	
	1st measurement	2nd measurement
EDE-Q		
*Restraint*		
rho	0.45	0.44
*P*	< 0.001	< 0.001
*Eating concern*		
rho	0.47	0.51
*P*	< 0.001	< 0.001
*Shape concern*		
rho	0.65	0.58
*P*	< 0.001	< 0.001
*Weight concern*		
rho	0.61	0.50
*P*	< 0.001	< 0.001
Global EDE-Q score		
rho	0.58	0.54
*P*	< 0.001	< 0.001

Table 5 shows Spearman's correlation coefficients (rho) between the patient's scores on the subscales of DBT-WCCL and the scores of the EDE-Q and BSL-23 at both measurements. In the first measurement, the "DBT Skills subscale" score of the DBT-WCCL was negatively correlated with all subscales and the global score of EDE-Q score and BSL-23 score. The better the patients' coping skills were in dealing with stressful situations, the fewer EDs they had, and the better they were in terms of BPD. Moreover, it was found that the "General Dysfunctional Coping Factor" subscale of the DBT-WCCL was positively correlated with the score of BSL-23 and with all the subscales and the total score of EDE-Q with the exception of the "Restraint dimension". This result indicates that dysfunctional coping in stressful situations was linked to more severe ED and BPD symptomatology. Finally, the "Blaming others factor" subscale score was not significantly correlated with any other measurement. The same results were observed in the 2nd measurement. Additionally, it was found that EDE-Q "Restraint dimension" was significantly correlated with DBT-WCCL general dysfunctional coping factor.

**Table 5:** Spearman's correlation coefficients (rho) between the patient’s scores on the subscales of DBT-Ways of Coping Checklist (DBT-WCCL) and the scores of the ED scale (EDE-Q) and the BPD scale (BSL-23)

	DBT-WCCL					
	1st measurement			2nd measurement		
	DBT Skills subscale	General dysfunctional coping factor	Blaming others factor	DBT Skills subscale	General dysfunctional coping factor	Blaming others factor
**EDE-Q**						
*Restraint*						
rho	− 0.28	0.13	− 0.18	− 0.39	0.38	− 0.02
*P*	0.018	0.265	0.138	0.001	0.001	0.867
*Eating concern*						
rho	− 0.31	0.26	− 0.16	− 0.42	0.34	− 0.12
*P*	0.009	0.025	0.167	< 0.001	0.003	0.327
*Shape concern*						
rho	− 0.28	0.29	− 0.01	− 0.37	0.42	0.09
*P*	0.017	0.014	0.946	0.001	< 0.001	0.448
*Weight concern*						
rho	− 0.30	0.28	− 0.07	− 0.31	0.36	0.05
*P*	0.011	0.016	0.578	0.008	0.002	0.689
Global EDE-Q score						
rho	− 0.32	0.26	− 0.12	− 0.40	0.42	0.01
*P*	0.006	0.026	0.326	0.001	< 0.001	0.939
**BSL-23**						
rho	− 0.35	0.40	0.09	− 0.51	0.51	0.07
*P*	0.003	0.001	0.434	< 0.001	< 0.001	0.586

Finally, the SBQ score was found to be positively correlated with the “Shape concern” and “Weight concern” subscales of the EDE-Q and the BSL-23 score. In the second measurement, all results of the first measurement remained significant. Additionally, it was found that greater SBQ was significantly associated with greater restraint and global EDE-Q score (Table 6).

**Table 6:** Spearman's correlation coefficients (rho) between participants' scores on the Suicidal Ideation Scale (SBQ) with ED Scale (EDE-Q), BPD Scale (BSL-23), and DBT-Ways of Coping Checklist (DBT-WCCL)

	SBQ	
	1st measurement	2nd measurement
EDE-Q		
*Restraint*		
rho	0.14	0.25
*P*	0.259	0.036
*Eating concern*		
rho	0.09	0.19
*P*	0.484	0.125
*Shape concern*		
rho	0.27	0.31
*P*	0.029	0.010
*Weight concern*		
rho	0.26	0.29
*P*	0.033	0.014
*Global EDE-Q score*		
rho	0.17	0.26
*P*	0.157	0.027
BSL-23		
rho	0.47	0.43
*P*	< 0.001	< 0.001
DBT-WCCL		
*DBT Skills subscale*		
rho	− 0.14	− 0.21
*P*	0.254	0.085
*General dysfunctional coping factor*		
rho	0.22	0.22
*P*	0.075	0.073
*Blaming others factor*		
rho	0.07	0.08
*P*	0.575	0.530

## Discussion

Skillful emotion regulation appears to be an important challenge for individuals diagnosed with BPD and EDs, and as a result, there has been a growing interest in researching novel therapies such as DBT that are putting an emphasis on observing, labeling and regulating emotion. The present study applied a 1-year DBT program to individuals with BPD and EDs. The dropout rate was 17% and no significant differences concerning demographics and questionnaires’ scores were found between the patients who abandoned therapy and those who completed the treatment. Among individual suffering from BPD a diagnosis of ED increases the probability of early treatment dropout [38], although socio-demographical factors and severity of BPD psychopathology do not seem to predict dropout from therapy [38, 39].

The first main finding of the study was the significant improvement in all outcome measures at post-treatment compared to those at pre-treatment. Specifically, after one year of DBT, the individuals showed a significant decrease in suicidal ideation, BPD, and ED symptomatology. It should be noted that although the sample size was relatively small all comparisons were highly significant. These results are supported also by previous studies that evaluated the effectiveness of DBT in individuals suffering from BPD and ED [25–29].

The second major finding was that BPD symptomatology was significantly related to ED symptomatology. The relationship between BPD symptoms and EDs is complex, and the mechanism underlying this comorbidity is still under investigation. According to some theoretical suggestions, the symptoms of BPD can act as a predisposing factor or a complication of EDs [4–6]. In order to receive a diagnosis of BPD, at least five of nine criteria must be met including: fear of abandonment, unstable interpersonal relationships, unstable self-image, impulsivity, self-harm, mood instability, feelings of emptiness, inappropriate anger, emptiness and dissociation/transient paranoid ideation [40]. The numerous possible combinations of BPD symptoms complicate our understanding of the mechanisms underlying comorbidity between BPD and ED, as the two disorders may be linked for different reasons depending on the BPD symptomatology. Certain BPD symptoms may play a greater role in comorbidity between ED and BPD than others contributing uniquely to the onset and manifestation of the disorder and to the course of treatment [14].

Recently, three studies have examined the nine individual symptoms of BPD in relation to the symptoms of EDs. Both, BN and AN symptoms, were more severe in an adolescent psychiatric inpatient sample who endorsed unstable interpersonal relationships, mood instability, feelings of emptiness, unstable self-image, inappropriate anger, dissociation/paranoia, and suicidal behavior while impulsivity was only related to BN symptoms [41]. Furthermore, emotional instability and anger were found to be significantly more strongly associated with symptoms of BN compared with AN, whereas the opposite was found for identity disturbance [41]. A network analysis for the visualization of the direct and indirect associations between transdiagnostic variables involved in BPD and ED symptoms revealed that emotion dysregulation and fear of abandonment are the most central symptoms in the relationship between BPD and EDs [16]. Finally, nine separate meta-analyses (one for each BPD symptom) which were conducted in order to compare levels of BPD symptoms in individuals with EDs versus healthy controls revealed that emotional instability was the most elevated BPD symptom, indicating that BPD patients may use maladaptive food-related behaviors to regulate intense negative emotions and rapid mood fluctuations [14].

These research results could lead to the assumption that DBT can be regarded as a promising treatment for individuals with BPD and EDs because it specifically targets deficits in emotion regulation by improving behavioral skills for distress tolerance, emotion regulation, and mindfulness [7, 20, 21]. The above suggestion is in accordance with the third finding of the study, that is, the improvement of the patients coping skills is correlated with the reduction of the ED and BPD symptomatology. These findings support the hypothesis that when individuals become more skillful in regulating their emotions they reduce the use of food as a way to "solve their problems" [15].

Future research is needed to explore which specific DBT skills may be more helpful for this clinical population. Randomized control studies are also needed to establish the efficacy of DBT for BPD and ED. Long-term follow-up studies could also examine or even confirm the long-term efficacy of this treatment and the factors that contribute to the remission of both disorders. Finally, a larger sample size would possibly indicate significant differences between different types of ED on outcome measures.

The study’s findings could probably have several clinical implications. Firstly, given the fact that most of the study’s participants applied only for ED treatment and the diagnosis of BPD was considered during their first assessment at the ED unit, it is essential to raise awareness among mental health practitioners of the comorbidity between ED and BPD and its clinical implications. Secondly, the correlation between BPD and ED symptomatology and the simultaneous improvement of both conditions after one year of DBT could indicate the value of addressing both disorders at the same time in order to achieve a more favorable treatment outcome. Finally, the low dropout rate (17%) could be regarded as a possible indication that participants found DBT an effective treatment option that could provide understanding, acceptance and a skillful way to restore their mental health.

In conclusion, it should be noted that the comorbidity of ED and BPD poses an ethical question to the clinician. Since the standard treatment for ED, such as CBT or family-based therapy, is not very helpful for severe cases of BPD and both conditions can prove dangerous for the sufferer's life, should a clinician focus on one of them first and then deal with the other using two different treatment plans? DBT for ED seems to provide an effective solution for this dilemma, providing a unified approach to the maladaptive behaviors related to both mental disorders. Of course, further research, primarily randomized controlled studies, is needed with a larger sample size in order to explore the effectiveness of DBT for this excessively burdened group of young sufferers.

### Strengths and limitations

The study has several strengths and limitations. It is the first study that evaluated a 1-year DBT intervention in individuals suffering from both BPD and EDs compared with previous studies that evaluated DBT interventions with shorter duration. The second strength is that both ED and BPD were addressed simultaneously as the treatment protocol dictated. Finally, the low dropout rate (17%), despite the fact that BPD comorbidity with ED typically increases the likelihood of dropout, enabled a degree of safety in the interpretation of the results.

However, the study also presents several limitations. The sample size was small and derived from a single treatment facility in the Greek capital’s central area. A control group was not included, as implementing any other form of effective treatment for this specific group of patients for an entire year posed significant challenges. Moreover, all AN and BN patients were grouped together, and BED patients were not included in the study. This hinders the ability to draw conclusions about the effectiveness of DBT in different diagnostic categories of EDs. Finally, another limitation is that confounding factors, such as depression and other anxiety disorders, were not assessed, although these factors are often present in individuals with personality disorders.

### What is already known on this subject?

DBT is an effective treatment for individuals suffering from BPD while promising results have also been found when ED’s patients were treated with DBT or DBT derived treatment protocols. Recently, there has been an attempt to apply DBT as a treatment for BPD and ED patients. The results from the published small number of open trials have been so far favorable for DBT.

### What this study adds?

The current study is the first to evaluate the effectiveness of a 1-year standard DBT treatment enhanced with CBT strategies for weight and nutritional restoration for patients suffering from ED and BPD. The findings of the study lead to the assumption that DBT could prove to be a promising treatment for individuals with BPD and EDs because it specifically targets deficits in emotion regulation by improving patients’ coping skills. Additionally, the analysis of the findings showed that improvement was simultaneously observed for both ED and BPD symptomatology and highly correlated with the enhancement of the patients’ behavioral skills.

### Supplementary Information

Below is the link to the electronic supplementary material.

Supplementary file1 (DOCX 10 KB)

## Data Availability

The datasets generated during and/or analysed during the current study are available from the corresponding author on reasonable request.
