# Supplementary material for: A 12-month study of dialectical behavioral therapy for bοrderline patients suffering from eating disorders
Source: Eat Weight Disord. 2023 Oct 5;28(1):81. doi: 10.1007/s40519-023-01612-w (PMC10556119; doi:10.1007/s40519-023-01612-w)
Supplement: Supplementary file 1 — Supplementary file1 (DOCX 10 KB) [file 40519_2023_1612_MOESM1_ESM.docx]

Supplementary Table: Comparison between the two ED diagnostic groups.

|  |  | 1st mearument | | 2nd measurement | |
| --- | --- | --- | --- | --- | --- |
|  |  | Mean | SD | Mean | SD |
| EDE-Q Restraint | Anorexia | 2.42 | 1.84 | 1.70 | 1.63 |
|  | Bulimia | 2.79 | 2.22 | 1.57 | 1.51 |
|  | P | 0.71 | | 0.867 | |
| EDE-Q Eating concern | Anorexia | 2.12 | 1.78 | 1.05 | 1.08 |
|  | Bulimia | 2.61 | 1.90 | 1.37 | 1.25 |
|  | P | 0.472 | | 0.388 | |
| EDE-Q Shape concern | Anorexia | 3.11 | 1.35 | 1.82 | 1.07 |
|  | Bulimia | 4.01 | 1.85 | 2.37 | 1.48 |
|  | P | 0.064 | | 0.283 | |
| EDE-Q Weight concern | Anorexia | 2.57 | 1.55 | 1.43 | 0.96 |
|  | Bulimia | 3.74 | 1.93 | 1.97 | 1.38 |
|  | P | 0.067 | | 0.217 | |
| Global EDE-Q | Anorexia | 2.55 | 1.42 | 1.50 | 1.10 |
|  | Bulimia | 3.29 | 1.82 | 1.82 | 1.34 |
|  | P | 0.179 | | 0.497 | |
| BSL score | Anorexia | 1.82 | 1.06 | 0.82 | 0.52 |
|  | Bulimia | 2.22 | 0.95 | 1.11 | 0.61 |
|  | P | 0.221 | | 0.116 | |
| WCCL DBT Skills subscale | Anorexia | 1.34 | 0.51 | 1.87 | 0.34 |
|  | Bulimia | 1.18 | 0.50 | 1.90 | 0.30 |
|  | P | 0.511 | | 0.445 | |
| WCCl General dysfunctional coping factor | Anorexia | 1.98 | 0.51 | 1.38 | 0.41 |
|  | Bulimia | 2.27 | 0.40 | 1.47 | 0.37 |
|  | P | 0.066 | | 0.431 | |
| WCCL Blaming others factor | Anorexia | 1.88 | 0.76 | 1.89 | 0.34 |
|  | Bulimia | 1.85 | 0.56 | 1.85 | 0.39 |
|  | P | 0.271 | | 0.795 | |

p-value for the difference between anorexia and bulimia nervosa
